# A framework for jointly modeling the natural history of ductal carcinoma in situ and invasive breast cancer

**DOI:** 10.1007/s00285-026-02418-x

**Published:** 2026-06-02

**Authors:** Evripidis Kapanidis, Keith Humphreys

**Affiliations:** 1 Department of Medical Epidemiology and Biostatistics, Karolinska Institute, SE-171 77 Solna, Sweden; 2 Swedish eScience Research Centre (SeRC), Linköping, Sweden

**Keywords:** Continuous Tumour Growth Model, DCIS, Breast Cancer Screening, Latent Processes, 62H12, 92-10

## Abstract

We present a new approach for jointly modelling the natural history of ductal carcinoma in situ (DCIS) and invasive breast cancer, based on a continuous tumor growth framework. We first describe the structure of a stable disease population, in which individuals traverse with rates invariant to calendar time through different health states. Based on the properties of this population we develop a likelihood model that jointly utilizes probability distributions describing sub-models for screening sensitivity, detection through symptoms, tumor growth rate and time for DCIS to become invasive. This model can incorporate any parametric forms for these sub-models, allowing for testing different assumptions for the occult biological progression of breast cancer. By using stable disease assumptions the model can be fitted to data from incident cancer cases and does not require specification of a sub-model for age at tumour onset. In the final part of the publication we perform simulations to verify the theoretical properties of the stable disease population and to show how our model can be used in a real setting to estimate characteristics of the involved latent processes.

## Introduction

Ductal Carcinoma in Situ (DCIS), is widely considered as a precursor of invasive ductal carcinoma. A DCIS lesion is comprised of abnormal cells, that are restricted in the ducts of the breast (Allred 2010). If left untreated, a portion of these lesions will, during the lifetime of an individual progress into invasive breast cancer (Wang et al. 2024).

The natural progression of DCIS to invasive breast cancer is not well understood and there are conflicting views regarding the proportion of DCIS lesions that will eventually transition to invasiveness (Erbas et al. 2006). Additionally, since the introduction of mammography screening to the general population, the incidence of DCIS has continuously increased throughout the decades (Kerlikowske 2010). Currently, it constitutes close to 20% of all newly diagnosed breast cancers (Parikh et al. 2018). Since the mechanism that causes invasion of the in situ lesion is not known, and the number of DCIS diagnoses has increased, there is a growing concern about overdiagnosis and overtreatment (Poelhekken et al. 2023).

Numerous statistical models have been developed in the past decades to describe the biological history of breast cancer. Specifically, multi-state Markov models utilizing data from trials or large observational studies have been widely used on prospective cohort data to infer parameters regarding time to developing breast cancer, disease progression and detection under mammography attendance (Duffy et al. 1995; Uhry et al. 2010). Alternatively, continuous growth models, which are based on the mathematical biology literature (Tsodikov and Yakovlev 1991; Bartoszyński et al. 2001) and jointly describe tumor growth, detection and screening attendance, have been developed for both prospective cohort data (Strandberg and Humphreys 2019; Weedon-Fekjær et al. 2010) and incident cases data (Isheden and Humphreys 2019). The main characteristic of using modeling approaches for data that includes only cases/diagnosed individuals (incident cases data), is that it does not require specification of a distribution for the age at breast cancer onset, but still allows estimating all the other parameters related to detection and growth of breast cancer. However, these approaches have been developed for invasive breast cancer only and there are currently no continuous growth models that simultaneously account for an in situ phase. Although many multi-state models (for prospective cohort data) have been extended to incorporate an in situ stage (Ravesteyn et al. 2018), they don’t allow for complex assumptions (e.g. transition rates dependent on size), which in turn restricts the available choices for quantitatively describing the natural history of DCIS. Additionally, the goodness of fit of multi-state models is mostly assessed through incidence rates of DCIS and cancer, and not in terms of continuous attributes, such as the tumor or lesion size distribution. Consequently, there is a need for developing new models, applicable under a wide range of different parametric assumptions, that will jointly model the growth of in situ lesions and their invasive components. Such models can then contribute to exploring and understanding the natural history of DCIS better.

The growth of DCIS lesions in isolation, has been previously modeled by Dowty et al. (2014). In their publication, they showed that under a number of assumptions, the distribution of DCIS sizes, which initially depends on calendar time, converges exponentially fast to a stationary pre-invasive size distribution. Based on these theoretical results and assuming a flexible two parameter power law growth function for DCIS growth, they used the stationary distribution and data on DCIS lesion size from a sample of 110 women diagnosed with DCIS to estimate the growth trajectories of DCIS lesions. However, there were some simplifying implicit assumptions in their approach; the modeling framework implied that healthy individuals could transition to latent DCIS but, from there, could only transition to invasive cancer, thus not taking into account the competing event of pre-invasive DCIS lesions becoming symptomatic. Moreover, the sample they used corresponded to screen detected women only, and their analysis indirectly implied an assumption of 100% screening sensitivity.

Describing a continuous tumour growth modelling framework which simultaneously incorporates competing risks of symptomatic detection of DCIS and progression to invasiveness, together with modelling of the competing risks of symptomatic and screen detection of both DCIS and invasive cancer, is challenging. We approach this by, like Dowty et al. (2014), using an approach based on stationary distributions. Stationary distribution approaches have also been developed in Isheden and Humphreys (2019), in that case based on the concept of the stable disease population for invasive breast cancer only. Specifically, they assumed that in the absence of screening, individuals transition through three health states, at rates independent of calendar time. Based on this, they then fitted a continuous tumor progression model to incident cases data. For their approach, the stable disease population constituted an essential assumption for successfully deriving the stationary distribution. A multi-state model for incident cases data was developed by Shen and Zelen (1999) some decades ago, who defined the stable disease population in the same way as Isheden and Humphreys (2019) and utilized its properties to estimate the model parameters. Although the likelihood contributions and the theoretical framework were different in these two papers, they made similar assumptions about the population of patients and both used a conditional likelihood approach, i.e. only data from individuals that were detected with breast cancer were used.

In this work, we develop the theoretical framework needed to include the DCIS stage as a part of the stable breast cancer population, by accounting for the competing events (progression to invasive cancer and symptomatic detection) that arise during this phase. Based on this new disease population, for incident cases data, we then develop a likelihood model for DCIS lesion size or invasive tumor size and the type of carcinoma (invasive or in situ), conditional on the screening history and overall mode of detection (symptomatic or screen detection). This requires information that is available from breast cancer registers. It also requires information on screening visits (timing of prior negative screens). In other studies this information has been obtained from local screening registers. In the present paper, we use the power law growth function that Dowty et al. (2014) used for DCIS lesions, although our modelling framework allows for any growth function to be used. In the final part of the paper we perform simulations to assess the identifiability of our model parameters and confirm the theoretical results that we derive.

## The stable disease population under competing events

### A continuous growth model for DCIS and invasive cancer

We begin by defining five states, in terms of which (along with DCIS lesion size and primary invasive cancer size) we will describe stable disease population results:

1. State before DCIS onset (Healthy)
2. State of preclinical pre-invasive state of DCIS
3. State of post-symptomatic detection of DCIS
4. State of preclinical invasive cancer
5. State of post-symptomatic detection of invasive cancer.

**Fig. 1:**
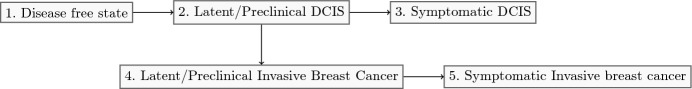
Representation of the five states upon which the continuous growth models are based

In our framework (represented graphically in Figure 1), we need to deal with the competing risks of transitioning to states 3 or 4 from state 2. Thus we represent time spent in state 2 by the minimum of the two random variables that correspond to transitioning to invasiveness and symptomatic detection. As in Isheden and Humphreys (2019, 2022) and in Dowty et al. (2014), we assume that the rate at which women enter and exit each state is independent of calendar time, which guarantees that the population is indeed stable and that all of the relevant distributions, such as time to symptomatic detection, are invariant with respect to time. In our work, calendar time refers to any type of chronological sequence, whose points, denoted as s, are also equidistant. For example, calendar time points could be seen as a sequence of dates 01/01/1990,02/01/1990,...., or as a more trivial sequence such as 0,0.01,0.02,..., where during each point in the sequence a fixed number of tumors is born (due to the stable disease population assumption). At each of these time points, the total population of people at risk including prevalent cases, is also assumed to remain stable. Note that the stable disease population in Isheden and Humphreys (2019, 2022) was defined only in terms of states $1 \rightarrow 4 \rightarrow 5$. The stable disease population of Dowty et al. (2014) was defined in terms of states $1 \rightarrow 2 \rightarrow 4$. As described later, we model growth of lesion/tumour sizes in states 2 and 4 using biologically motivated models, which distinguishes the models from classical multi-state models.

### Theoretical results for the stable disease population

In this section, we derive some theoretical properties that directly stem from the assumption of having a stable disease population that does not attend screening. In section 4, we show how these properties can be easily combined with the screening patterns and tumor measurements of individuals to derive their whole likelihood contributions. Thus, the theoretical results presented here are fundamental to the practical applications of the model. A brief definition of each random variable defined in the subsections of 2.2, can be found in table 1.

**Table 1:** Variable Names And Definitions

Parameters	Definition
$T_{D}$	time from onset of DCIS to DCIS (D) symptomatic detection
$T_{T}$	time from DCIS onset to transitioning (T) to invasive cancer
$T_{I}$	time from onset of invasive (I) cancer to symptoms
$T_{D}(s)$	time spent from onset of DCIS until timepoint s
$T_{I}(s)$	time spent from onset of invasive cancer until timepoint s

#### Probabilities of preclinical state membership and transitions to symptomatic detection

Below we provide formulas for the probabilities of being in the DCIS and invasive preclinical states (states 2 and 4, respectively). In lemma 2 of Isheden and Humphreys (2022), which was based on the results of Freeman and Hutchison (1980), and modeled the disease population as consisting of states 1,4 and 5, it was shown that in a population defined over a large window of calendar time, the conditional probability (conditional on sojourn time *t*) for an individual to have a preclinical cancer at a particular time point *s* (within the calender time window), is proportional to *t*. For this 3 state model this was written as $P(A(s)=1|T_{det}=t)\propto t$ in Isheden and Humphreys (2022), where $A(s)=1$ denotes being in the preclinical state at calender time *s* and $T_{det}$ is the time spent in the preclinical state. This result holds in the absence of competing risks. Since this result can be seen as a starting point for the current paper, we explain it in detail in Appendix A. As Isheden and Humphreys (2022) worked with this conditional probability in e.g. derivations of likelihoods for the model consisting of states 1, 4 and 5, above, we work with similar conditional probabilities in derivations presented herein for the model with DCIS and invasive breast cancer. Now, since, states 1,2 and the combination of states 3 and 4 can be viewed as comprising a stable DCIS subpopulation, the following lemma, which constitutes a simple modification of lemma 2 in Isheden and Humphreys (2022) (or equivalently lemma 1 in Isheden and Humphreys (2019)), will hold:

##### Lemma 1

Let $A_{1}(s)$ be a random variable that is defined as follows:

$$ A_{1}(s)= \left\{ \begin{array}{ll} 1, & \text {At calendar time s the patient is at the DCIS preclinical state}\\ 0, & \text {At calendar time s the patient is not at the DCIS preclinical state}\\ \end{array} \right. $$

Let also $T_{D},T_{T}$ be the random variables that denote the time it takes, from entering state 2, to develop DCIS symptoms and to transition to invasive cancer, respectively. It then holds that:

$$\begin{aligned} P(A_1(s)=1|min\{T_{D},T_{T}\}=t)\propto t \end{aligned}$$

That is, the probability of being in the preclinical DCIS state should be proportional to its sojourn time which in this case is the minimum between the time to symptomatic detection and transitioning to insasive cancer.

Similarly, we can derive a formula for the probability of having progressed to invasive cancer and being in the latent invasive state at calendar time point s:

##### Lemma 2

Let $A_{2}(s)$ be a random variable that is defined as:

$$ A_{2}(s)= \left\{ \begin{array}{ll} 1, & \text {At calendar time s the person is in a preclinical invasive state}\\ 0, & \text {At calendar time s the person is not in a preclinical invasive state}\\ \end{array} \right. $$

Then if $T_{I}$ denotes the time to symptomatic detection of invasive cancer, since entry to state 4, it holds that:

$$ P(A_{2}(s)=1|T_{I}=t_{i})\propto \frac{t_{i}f_{T_{I},T_{D}>T_{T}}(t_{i},1)}{f_{T_{i}}(t_{i})} $$

##### Proof

The variable $A_{2}(s)$ denotes the event of being in the latent invasive cancer state while accounting for the competing events before progression to invasive cancer. In contrast, we can introduce a random variable $A'_{2}(s)$ that refers only to the event of being in the latent invasive cancer state under elimination of the competing risk of symptomatic DCIS, in which case

$$ A_{2}(s)=A'_{2}(s)\cap \{T_{D}>T_{T}\} $$

and then using Bayes’ theorem we can write

1 $$\begin{aligned} \begin{aligned} P(A_{2}(s)=1|T_{I}=t_{i})&=\frac{f_{A_{2}(s),T_{I}}(1,t_{i})}{f_{T_{I}}(t_{i})}=\frac{\int _{0}^{+\infty }f_{A_{2}(s),T_{I},T_{T}}(1,t_{i},t_{t})dt_{t}}{f_{T_{I}}(t_{i})}\\&=\frac{ \int _{0}^{+\infty }f_{A'_{2}(s),T_{D}>T_{T} T_{I},T_{T}}(1,1,t_{i},t_{t})dt_{t}}{f_{T_{I}}(t_{i})} \end{aligned} \end{aligned}$$

Denoting as *N*(.) the number of people that satisfy the event inside the parentheses, we write:

$$ N( A_{2}(s)=1,T_{I}=t_{i},T_{T}=t_{t})=N(A'_{2}(s)=1,T_{I}=t_{i},T_{T}=t_{t},T_{T}<T_{D}) $$

It can then be seen that the total number of people that have the potential to satisfy the event above are the ones that had their DCIS onset at calendar times $s-t_{t},s-1-t_{t},...,s-t_{i}+1-t_{t}$. If someone had an onset outside of these calendar times then certainly the conditions above would not be satisfied. Note that the number of possible calendar times is always equal to $t_{i}$. Thus if *I* is the number of new onsets per calendar time we have $t_{i}I$ as the total number of people that could satisfy the event. From all of these people the proportion of those that also satisfy $\{T_{I}=t_{i},T_{T}=t_{t},T_{T}<T_{D}\}$ is represented by the individuals that do indeed satisfy the whole event. thus we can write in total:

2 $$\begin{aligned} \begin{aligned}&N(\{ A'_{2}(s)=1,T_{I}=t_{i},T_{T}=t_{t},T_{T}<T_{D}\})=t_{i}\times I \times f_{T_{I},T_{T},T_{T}<T_{D}}(t_{i},t_{t},1) \end{aligned} \end{aligned}$$

Dividing then by the total population size *N*, we get the density in the integrand, so that:

3 $$\begin{aligned} P(A_{2}(s)=1,T_{I}=t_{i})=\int _{0}^{+\infty }t_{i}\times \frac{I}{N} \times f_{T_{I},T_{T},T_{T}<T_{D}}(t_{i},t_{t},1)dt_{t}= t_{i}\frac{I}{N}f_{T_{I},T_{D}>T_{T}}(t_{i},1) \end{aligned}$$

which concludes the proof of Lemma 2. $\square$

The following theorem concerns the transitions to the symptomatic detection states:

##### Theorem 1

If

$$ D_{2}(s)= \left\{ \begin{array}{ll} 1, & \text {Symptomatic detection of invasive cancer at s}\\ 0, & \text {No symptomatic detection of invasive cancer at s}\\ \end{array} \right. $$

$$ D_{1}(s)= \left\{ \begin{array}{ll} 1, & \text {Symptomatic detection of DCIS at s}\\ 0, & \text {No symptomatic detection of DCIS at s}\\ \end{array} \right. $$

then:

$$ f_{D_{2}(s),T_{I} }(1,t_{i})\propto f_{T_{T}<T_{D},T_{I}}(1,t_{i}) $$

and

$$ f_{D_{1}(s),T_{D} }(1,t_{d})\propto f_{T_{T}<T_{D},T_{D}}(0,t_{d}) $$

##### Proof

Similar to lemma 2, $D_{1}(s),D_{2}(s)$ denote the events of being symptomatically detected at timepoint s with DCIS or invasive cancer respectively, accounting for the competing risks of state 2. By introducing the random variables $D'_{1}(s),D'_{2}(s)$, which correspond to being symptomatically detected with DCIS or invasive breast cancer under elimination of the competing events in state 2, we can write:

$$ D_{2}(s)=D'_{2}(s)\cap \{ T_{T}<T_{D} \} $$

$$ D_{1}(s)=D'_{1}(s)\cap \{ T_{T}>T_{D} \} $$

For the symptomatic detection of invasive cancer, marginalizing with respect to $T_{T}$ yields:

4 $$\begin{aligned} \begin{aligned} f_{D_{2}(s),T_{I}}(1,t_{i})=\int _{0}^{+\infty }f_{D_{2}(s),T_{I},T_{T}}(1,t_{i},t_{t})dt_{t}=\int _{0}^{+\infty }f_{D'_{2}(s),T_{D}>T_{T}, T_{I},T_{T}}(1,1,t_{i},t_{t})dt_{t} \end{aligned} \end{aligned}$$

Using a similar framework as in lemma 2, the proportion of cancers that have an onset exactly at $$s-(T_{I}+T_{T})$$ and that also satisfy $$ T_{I}=t_{i},T_{T}=t_{t}, T_{D}>T_{T}$$ is equal to the joint distribution in the integrand of (4). Thus we can write:

5 $$\begin{aligned} \begin{aligned} f_{D_{2}(s),T_{I}}(1,t_{i})=\int _{0}^{+\infty }\frac{I}{N} f_{T_{D}>T_{T},T_{T}, T_{I}}(1,t_{t},t_{i})dt_t =\frac{I}{N} f_{T_{D}>T_{T}, T_{I}}(1,t_{i}) \end{aligned} \end{aligned}$$

The proof for the DCIS symptomatic detection follows immediately by noting that the cancer should have its onset at timepoint $s-T_{D}$. $\square$

Finally we consider the (stationary) conditional probabilities of membership of the preclinical states:

##### Lemma 3

It generally holds that:

$$\begin{aligned} \begin{aligned} P(A_{1}(s)=1|\{A_{1}(s)\cup A_{2}(s)\}=1)=\frac{E(T_{e})}{E(T_{e})+\int _{0}^{+\infty }t_{i}f_{T_{I},T_{D}<T_{T}}(t_{i},0)dt_{i}} \end{aligned} \end{aligned}$$

where $T_{e}=min(T_{T},T_{D})$, the minimum exiting time from the DCIS latent stage.

##### Proof

The result follows by noting that:

$$\begin{aligned} \begin{aligned} P(A_{1}(s)=1)=\frac{I}{N}\int _{0}^{+\infty }t_{e}f_{T_{e}}(t_{e})dt_{e}=\frac{I}{N}E(T_{e}) \end{aligned} \end{aligned}$$

$$\begin{aligned} \begin{aligned} P(A_{2}(s)=1)=\frac{I}{N}\int _{0}^{+\infty }t_{i}f_{T_{I},T_{D}<T_{T}}(t_{i},0)dt_{i} \end{aligned} \end{aligned}$$

$$\square $$

#### Distributions of times since entry to preclinical states

In this subsection we provide properties regarding the distributions of times so far spent in the preclinical states at a calendar time point *s*.

##### Lemma 4

If $T_{D}(s)$ is a random variable that corresponds to time since entry to state 2 at calendar time s, then it holds that :

$$\begin{aligned} P(T_{D}(s)=t'|A_{1}(s)=1,min\{T_{D},T_{T}\}=t)\overset{D}{=}\ U(0,t) \text {, when } t>t'>0 \end{aligned}$$

Lemma 4 constitutes a simple modification of the theorem presented in Isheden and Humphreys (2019, 2022) and thus the proof is omitted.

##### Lemma 5

If $T_{I}(s)$ is a random variable that corresponds to time since transition to invasive cancer (i.e. entry to state 4) at calendar time s, then it holds that:

$$\begin{aligned} P(T_{I}(s)=t'|A_{2}(s)=1,T_{I}=t_{i})\overset{D}{=}U(0,t_{i}) \text {, when } t_{i}>t'>0 \end{aligned}$$

##### Proof

Expanding the conditional probability using Bayes’ theorem the numerator can be written as:

6 $$\begin{aligned} \begin{aligned} f_{T_{I}(s),A_{2}(s),T_{I}}(t',1,t_{i})=\int _{0}^{+\infty } f_{T_{I}(s),A'_{2}(s),T_{I},T_{T},T_{T}<T_{D}}(t',1,t_{i},t_{t},1)dt_{t} \end{aligned} \end{aligned}$$

where $t'<t_{i}$. Note that $A'_{2}(s)$ becomes redundant in the integrated joint distribution of (6) since $0<t'<t_{i}$. Defining the random variable $T_{Onset}$ as the calendar time of entry to state 2, and noting that when $T_{I}(s)>0$, we can write:

$$ T_{I}(s)=s-(T_{T}+T_{Onset}) $$

Entry to state 2 should have then taken place at calendar time $s-t'-t_{t}$. $T_{I}(s)$ can then be replaced by $T_{Onset}$ through a simple random variable transformation. Thus:

7 $$\begin{aligned} \begin{aligned}&\int _{0}^{+\infty }f_{T_{I}(s),A_{2}(s),T_{I},T_{T}}(t',1,t_{i},t_{t})dt_{t}=\int _{0}^{+\infty }f_{T_{Onset},T_{I},T_{T},T_{T}<T_{D}}(s-t'-t_{t},t_{i},t_{t},1)dt_{t}\\&=\int _{0}^{+\infty }\frac{I}{N}\times f_{T_{I},T_{T},T_{T}<T_{D}}(t_{i},t_{t},1)dt_{t}=\frac{I}{N}f_{T_{I},T_{T}<T_{D}}(t_{i},1) \end{aligned} \end{aligned}$$

Consequently the conditional probability can be written as a whole as:

8 $$\begin{aligned} \begin{aligned} P(T_{I}(s)=t'|A_{2}(s)=1,T_{I}=t_{i})=\frac{f_{T_{I}(s),A_{2}(s),T_{T}}(t',1,t_{t})}{ f_{A_{2}(s),T_{I}}(1,t_{i}) }=\frac{I}{N} \frac{f_{T_{I},T_{T}<T_{D}}(t_{i},1)}{ f_{A_{2}(s),T_{I}}(1,t_{i}) } \end{aligned} \end{aligned}$$

Using the result of lemma 2 for the denominator the result follows immediately. $\square$

### Results under proportional hazards

Results in section 2.2 are derived without any assumption concerning the transition rates between states. In this subsection we explore the implications of this assumption and how it simplifies further the results of section 2.2.

Specifically, an interesting result that occurs assuming proportional hazards for $h_{D}(t), h_{T}(t),$ is that the probability of developing DCIS symptoms before progressing to invasive breast cancer, is constant:

#### Theorem 2

If $\frac{h_{T_{D} }(t)}{h_{T_{T}}(t)}=c$, where *c* is a constant, then:

$$\begin{aligned} P(T_{D}<T_{T})=c/(1+c) \end{aligned}$$

#### Proof

Marginalizing with respect to $T_{T}$ yields:

$$\begin{aligned} \begin{aligned} P(T_{D}<T_{T})&=\int _{0}^{+\infty } P(t_{d}<T_{T}|T_{D}=t_{d})f_{T_{D}}(t_{d})dt_{d}\\&=c\int _{0}^{+\infty }\exp ({-H_{T_{T}}(t_d)})h_{T_{T}}(t_{d})\exp ({-cH_{T_{T}}(t_d)})dt_{d}\\&=-\frac{c}{1+c}(\exp ({-(1+c)H_{T_{T}}(+\infty )})-\exp ({-(1+c)H_{T_{T}}(0)} ))=\frac{c}{1+c} \end{aligned} \end{aligned}$$

$$\square $$

Under proportional hazards, lemma 2 simplifies as follows:

#### Corollary 1

Under proportional hazards:

$$\begin{aligned} \begin{aligned} P(A_{2}(s)=1|T_{I}=t_{i})\propto t_{i} \end{aligned} \end{aligned}$$

#### Proof

The proof follows immediately by using the result of lemma 2. $\square$

Under proportional hazards, lemma 3 simplifies as follows:

#### Corollary 2

Under proportional hazards , i.e. $P(T_{D}<T_{T})=c/(1+c)=a$ :

$$\begin{aligned} \begin{aligned} P(A_{1}(s)=1|\{A_{1}(s)\cup A_{2}(s)\}=1)=\frac{E(T_{e})}{E(T_{e})+(1-a)E(T_{I})} \end{aligned} \end{aligned}$$

#### Proof

The results follow by noting that under proportional hazards:

$$\begin{aligned} \begin{aligned} P(A_{2}(s)=1)=\frac{I(1-a)}{N}\int _{0}^{+\infty }t_{i}f_{T_{I}}(t_{i})dt_{i} \end{aligned} \end{aligned}$$

The equality for the invasive component follows from lemmas 3,4,5. $\square$

## Parametric assumptions

Before proceeding to the likelihood derivation, we briefly present the parametric assumptions we make in our model’s components. We recognize that the parametric assumptions might need to be changed in order to capture breast cancer incidence well. Choices here are based broadly on other published papers (e.g. Dowty et al. (2014)) and serve sufficiently well for the purpose of illustrating our proposed modeling framework. Throughout our modelling approach we define symptomatic detection as a consequence of symptoms, that is, because a woman can feel a lump in her breast or because she has some other symptoms such as swelling of part of the breast or a lump in the armpit. In contrast screen detection corresponds to detection through regular screening mammography, when the tumor has not been diagnosed through other means.

Thomson et al. (2001), reported that DCIS lesions can grow in all directions, but that they grow fastest in a direction towards and away from the nipple. The maximum diameter of DCIS, which should correspond to the size in the fastest direction, may therefore constitute a good indicator/approximation of its overall size. We use the same growth function as Dowty et al. (2014) to represent the growth/size of DCIS lesions, namely the two parameter power law growth function. We use *R* to denote an individual-specific (inverse) growth rate, which here we assume follows a Gamma distribution $G(\tau _{1},\tau _{2})$, and we use *t* to denote the time elapsed since the onset of the lesion. Thus, if time *t* since onset has passed, a lesion of growth rate $R=r$ has size

$$\begin{aligned} l_{r}(t)=\Bigl ( \frac{kt(1-\beta )}{r}+l_{0}^{1-\beta }\Bigl )^{\frac{1}{1-\beta }} , \end{aligned}$$

where $k>0$ and $l_0$ is a (fixed) initial size at onset, which we set here to 0.03 mm. Regarding the mode of detection for in situ lesions, most of them are found by mammography, and specifically through the presence of visible microcalcifications in the mammogram (Holland and Hendriks 1994). Mammographic detection of non calcified DCIS, is also possible, although more rare, and is based on the presence of an abnormal mass or architectural distortion on the mammogram (Bragg et al. 2021). Screen detection of DCIS accounts for approximately 85 % of all DCIS diagnoses (Ernster et al. 2002). The rest of them are detected symptomatically, the symptoms usually being a palpable mass in the breast and nipple discharge (Shin et al. 2008). On average symptomatic DCIS lesions are larger than screen detected lesions (Koh et al. 2015). Additionally, it is less common for symptomatic DCIS lesions, than for screen detected lesions, to have visible calcifications in previous mammography examinations (Bragg et al. 2021; Cho et al. 2008). While recognizing the large heterogeneity of DCIS, we base our parametric assumptions on the idea that, broadly speaking, the progression to invasive cancer as well as the detection of in situ lesions is attributed to their size. Specifically, we assume that the probability of being screen detected with DCIS, when attending a screen whilst in state 2, given a current lesion size *l*, is:

$$\begin{aligned} \frac{\exp ({b_{0}+b_{1}l })}{1+\exp ({b_{0}+b_{1}l })} \end{aligned}$$

Our choice is based on the intuition that as the lesion grows, so does the extent of the microcalcifications or the mass in the mammogram, making it easier to detect. We specifically use the logistic function because of its flexibility, but other functions are possible. We also consider the hazards for DCIS symptomatic detection and transition to invasive cancer as being proportional to size, i.e. :

$$\begin{aligned} h_{T_{D}}(t|r)=\eta _{D}l_{r}(t)=\eta _{D} \Bigl ( \frac{kt(1-\beta )}{r}+l_{0}^{1-\beta }\Bigl )^{\frac{1}{1-\beta }}, \,\eta _{D}>0 \end{aligned}$$

$$\begin{aligned} h_{T_{T}}(t|r)=\eta _{T}l_{r}(t)=\eta _{T} \Bigl ( \frac{kt(1-\beta )}{r}+l_{0}^{1-\beta }\Bigl )^{\frac{1}{1-\beta }},\, \eta _{T}>0 \end{aligned}$$

Support for the progression to invasive cancer model comes from studies using MRI that have reported that the size of the DCIS is associated with an elevated risk of microinvasion (Miceli et al. 2023; Lamb et al. 2020). Note that, under these parametric assumptions, conditional on *R*, the hazards are still proportional and through the use of theorem 2 it holds directly that:

$$\begin{aligned} P(T_{D}<T_{T}|R=r)=\frac{c}{1+c} \implies P(T_{D}<T_{T})=\frac{c}{1+c} \end{aligned}$$

For the invasive component, we consider an exponential growth function which has been used in previous publications (Strandberg and Humphreys 2019; Isheden and Humphreys 2019; Abrahamsson and Humphreys 2016). For tumour volume, with tumours assumed to be spherical, the growth function is denoted as $V_{R}(t)=v_{0}\exp (t/R)$, *R* being the (inverse) growth rate, *t* the time since the initiation of the invasive tumour, and $v_{0}$ an initial size constant, which in this paper we have set to 0.05 $\text {mm}^{3}$. To reduce the computational complexity of our approach, we assume that the DCIS and invasive growth rates of each individual, which constitute a measure of disease aggressiveness, are equal. Studies have reported that biological characteristics (e.g. grade) associated with the aggressiveness of in situ lesions will be passed on to the resulting invasive component (Van Luijt et al. 2016; Cadman et al. 1997). For assuming different, but correlated, growth rates in DCIS lesions and invasive cancers, within individuals, see the Discussion section.

For the screening sensitivity of invasive breast cancer we use the logistic model as a function of the invasive tumour diameter, and the accompanying DCIS component. Assuming *d* is the diameter of the invasive tumor and *l* is the size of the in situ component, the screening sensitivity for invasive cancer, when attending a screen whilst in state 4, given a current invasive tumour diameter, *d*, and DCIS lesion size, *l*, is given by:

$$\begin{aligned} \frac{\exp ({b_{0}+b_{1}l+b_{2}d })}{1+\exp ({b_{0}+b_{1}l+b_{2}d })} \end{aligned}$$

The logistic model has been previously used in publications for modelling the natural history of invasive breast cancer (Weedon-Fekjær et al. 2010; Strandberg and Humphreys 2019; Isheden and Humphreys 2019; Abrahamsson and Humphreys 2016). We include the DCIS component in the above detection probability to reflect on previous reports that approximately 20 percent of screen detected in situ lesions are upgraded to invasive cancer after the final pathology, i.e. a proportion of reported invasive breast cancers are detected because of the in situ component (Mayfield et al. 2024). For the symptomatic detection of the invasive cancer we express its hazard function, given the tumour growth rate, as being dependent on the tumour size:

$$\begin{aligned} h_{T_{I}}(t|r)=\eta _{I}V_{r}(t)=\eta _{I}v_{0}\exp (t/r) \end{aligned}$$

This hazard function has been widely used in previous continuous tumor progression modelling publications (Isheden and Humphreys 2019; Strandberg and Humphreys 2019; Abrahamsson and Humphreys 2016; Plevritis et al. 2007). Through this assumption the distribution of tumor sizes, $V_{I}$, at symptomatic detection, i.e. in the absence of screening is:

$$\begin{aligned} f_{V_{I}}(v)=\int _{0}^{+\infty }f_{V_{I}|R}(v|r)f_{R}(r)dr \end{aligned}$$

$$\begin{aligned} =v^{-1}\int _{0}^{+\infty }rh_{T_{I}|R}\left( rln(\frac{v}{v_{0}})|r\right) \exp ({-H_{T_I|R}\left( rln(\frac{v}{v_{0}})|r\right) })f_{R}(r)dr \end{aligned}$$

which can be shown to reduce to:

$$\begin{aligned} f_{V_{I}}(v)=\tau _1\tau _2^{-1}\eta _{I} \mathcal {F}\{\eta _{I}(v-v_{0}) \} \end{aligned}$$

where $\mathcal {F}$ is the Laplace transform of $G(\tau _{1}+1,\tau _{2})$. Note that the above density corresponds to a distribution of symptomatic sizes without accounting for the competing risks, which would correspond to writing $f_{V_{I}|T_{D}>T_{T}}(v|1)$. However, under the proportional hazards assumption, it can be easily shown that the analytic formula for the symptomatic sizes will remain the same, when accounting for competing events. Similarly under the assumptions of this section, defining $f_{L_{D}}$ as the distribution of DCIS lesion sizes at symptomatic detection, i.e. under no screening, following the same procedure yields:

$$\begin{aligned} f_{L_{D}}(l)=\int _{0}^{+\infty }f_{L_{D}|R}(l|r)f_{R}(r)dr=\tau _1\tau _2^{-1}\eta _{D}l^{1-b}k^{-1}\mathcal {F}\bigg \{\eta _{D} \frac{ l^{2-\beta }-l_{0}^{2-\beta } }{k(2-\beta )} \bigg \} \end{aligned}$$

Also, the distribution of symptomatic DCIS sizes while accounting for the competing risks, under proportional hazards, is:

$$ f_{L_{D}|T_{D}<T_{T}}(l|1)=c^{-1}(1+c)\tau _1\tau _2^{-1}\eta _{D}l^{1-b}k^{-1}\mathcal {F}\bigg \{(\eta _{D}+\eta _{T}) \frac{ l^{2-\beta }-l_{0}^{2-\beta } }{k(2-\beta )} \bigg \} $$

## Likelihood contributions

In this section, we derive the likelihood contributions for our model under the assumption and properties of the stable disease population. As previously noted, our theoretical and methodological results make it possible to fit our model to data collected using a cases-only design, i.e. to data that contains only individuals that have been detected with invasive or in situ carcinoma within a specific time interval (e.g. of several years). We describe a likelihood function that has contributions that are conditional on the mode of detection and the timing of prior negative screens. To improve the identifiability of the model we do not only model lesion/tumour size, but we also (jointly) model the disease type (DCIS/invasive). For likelihood contributions that correspond to the detection of invasive cancers we do not rely on information for the size of the DCIS component. We derive the likelihood contributions for general functions describing the transitions from pre-clinical DCIS (to either DCIS symptomatic detection or pre-clinical invasive cancer), but describe how simplifications occur under proportional hazards. Before describing each likelihood contribution analytically, we define the random variable:

$$\begin{aligned} B(s)= \left\{ \begin{array}{ll} 1, & \text {Cancer detected on mammogram when a woman attends screening at calendar point} s\\ 0, & \text {Cancer not detected on mammogram when a woman attends screening at calendar point} s\\ \end{array} \right. \end{aligned}$$

We also define the random variables $A(s)=A_{1}(s)\cup A_{2}(s)$ and $D(s)=D_{1}(s)\cup D_{2}(s)$.

We use ${\textbf {B}}$ to denote the previous screening history of individuals. The probability of screen detection is considered to be independent of that of the previous screenings, after conditioning on tumor or lesion sizes at each screen. In the following four subsections we derived likelihood contributions for four types of detection. Notations used for variables are the same as those described in Sect. 3.

### Likelihood contribution of screen detected invasive cancer

The likelihood contribution of this type of case can be written as:

9 $$\begin{aligned} \begin{aligned}&f_{A_{2}(s),V(s)| B(s),\textbf{B}, A(s) }(1,v|1,\textbf{0},1)\propto f_{A_{2}(s),V(s),B(s),\textbf{B}}(1,v,1,\textbf{0})\\ &=\int _{0}^{+\infty } \int _{0}^{+\infty } P(B(s)=1,\textbf{B}=\textbf{0}|R=r,T_{T}=t_{t},V(s)=v,A_{2}(s)=1)\times \\&\quad \times f_{ T_{T},A_{2}(s),V(s),R}(t_{t},1,v,r)drdt_{t} \end{aligned} \end{aligned}$$

**Fig. 2:**
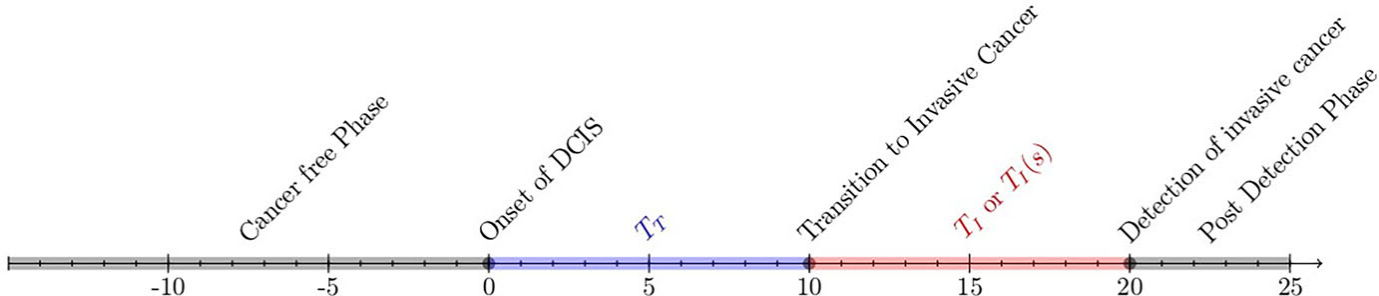
An example timeline showcasing disease progression for a person who was detected with invasive cancer. Integrating with respect to *R* and knowing the tumor volume we can calculate $T_{I}(s)$ or $T_{I}$ (depending on the mode of detection) and by integrating w.r.t. $T_{T}$ we can estimate the sizes of both DCIS and invasive cancers at any timepoint. Thus we can estimate the screening sensitivities at each examination. If an examination took place before the onset of DCIS (before 0) its sensitivity is 0

Conditional on $R,T_{T}, V(s)$, one can back-project to find the values of the invasive tumors and lesion sizes during the previous screenings (see Figure 2). The joint distribution of $T_{T},A_{2}(s),V(s),R$ can be derived as:

10 $$\begin{aligned} \begin{aligned}&f_{ T_{T},A_{2}(s),V(s),R}(t_{t},1,v,r)=\int _{rln(\frac{v}{v_{0} })}^{+\infty }f_{ T_{T},A_{2}(s),V(s),R,T_{I}}(t_{t},1,v,r,t_{i})dt_{i}\\&=\int _{rln(\frac{v}{v_{0} })}^{+\infty }f_{V(s)| T_{T},A_{2}(s),R,T_{I}}(v|t_{t},1,r,t_{i})P(A_{2}(s)=1|t_{t},t_{i},r)f_{T_{I}|R}(t_{i}|r)f_{T_{T}|R}(t_{t}|r)f_{R}(r)dt_{i}\\&=\int _{rln(\frac{v}{v_{0} })}^{+\infty } rv^{-1}f_{T_{I}(s)| T_{T},A_{2}(s),R,T_{I}}(rln\left( \frac{v}{v_{0}}\right) |t_{t},1,r,t_{i})\frac{I}{N} \frac{t_i f_{T_D>T_I,T_T,T_I,R}(1,t_t,t_i,r)}{f_{T_T,T_I,R}(t_t,t_i,r)} \\&\quad \,\, \times f_{T_{I}|R}(t_{i}|r)f_{T_{T}|R}(t_{t}|r)f_{R}(r)dt_{i}\\&=rv^{-1}{t_i}^{-1} \frac{I}{N} t_i P_{T_{D}|R} (T_{D}>t_{t}|r) f_{T_{T}|R}(t_{t}|r)f_{R}(r)\int _{rln(\frac{v}{v_{0} })}^{+\infty }f_{T_{I}|R}(t_{i}|r)dt_{i}\\&=rv^{-1}\frac{I}{N}P_{T_{D}|R,T_{T} } (T_{D}>t_{t})|r,t_{t})f_{T_{T}|R}(t_{t}|r)f_{R}(r) P(T_{I}>rln(\frac{v}{v_{0} })|R=r) \end{aligned} \end{aligned}$$

Where the second from last equality occurred by directly applying lemma 4. The resulting expression inside the double integral can then be computed numerically. After performing some additional algebraic calculations the previous joint distribution can also be written as:

11 $$\begin{aligned} \begin{aligned}&f_{T_{T},A_{2}(s),V(s),R}(t_{t},1,v,r)\\&=\frac{I}{N} r v^{-1} P(T_{I}>rln(\frac{v}{v_{0}})|R=r) P(T_D>t_t|R=r,T_T=t_t)f_{T_{T}|R}(t_{t}|r)f_{R}(r)\\&= \frac{I}{N}rv^{-1} \frac{f_{T_{I}|R}(rln(\frac{v}{v_{0}})|r) f_{T_D>T_T,R,T_T}(1,r,t_t)}{h_{ T_{I}|R}(rln(\frac{v}{v_{0}})|r)}=\frac{I}{N} \frac{f_{V_{I}|R}(v|r)f_{T_D>T_T,T_T|R}(1,t_t|r)f_R(r)}{\eta _Iv} \\&=\frac{I}{N}\frac{f_{V_{I},T_D>T_T,T_{T},R}(v,1,t_{t},r)}{\eta _Iv} \end{aligned} \end{aligned}$$

Then, based on (11), if the proportionality of hazards assumption holds, the distribution of latent preclinical tumor sizes can be derived in the simple form below:

12 $$\begin{aligned} \begin{aligned} f_{V(s)|A_{2}(s)}(v|1)\propto \int _{0}^{+\infty } \int _{0}^{+\infty } f_{V(s),A_{2}(s),R,T_{T}}f(v,1,r,t_{t})drdt_{t} \propto \frac{f_{V_{I}}(v)}{v} \end{aligned} \end{aligned}$$

### Likelihood contribution of symptomatically detected invasive cancer

This likelihood contribution will be of the form:

13 $$\begin{aligned} \begin{aligned}&f_{D_{2}(s),V(s)| \textbf{B},D(s) }(1,v|\textbf{0},1)\propto f_{D_{2}(s),V(s),\textbf{B}}(1,v,\textbf{0})\\&=\int _{0}^{+\infty } \int _{0}^{+\infty } P(\textbf{B}=\textbf{0}|R=r,T_{T}=t_{t},V(s)=v,D_{2}(s)=1)f_{ T_{T},D_{2}(s),V(s),R}(t_{t},1,v,r)drdt_{t} \end{aligned} \end{aligned}$$

The joint density of $T_{T},D_{2}(s),V(s),R$ can be written as:

14 $$\begin{aligned} \begin{aligned}&f_{ T_{T},D_{2}(s),V(s),R}(t_{t},1,v,r)=f_{ V(s)|T_{T},D'_{2}(s),T_{D}<T_{T},R}(v|t_{t},1,0,r)f_{T_{T},D'_{2}(s),T_{D}<T_{T},R}(t_{t},1,0,r) \end{aligned} \end{aligned}$$

Then using Theorem 1 and the fact that $f_{V(s)|D'_{2}(s)}(v|1)=f_{V_{I}}(v)$, where $V_{I}$ is the tumor volume at symptomatic detection, we can rewrite the formula in (14) as:

15 $$\begin{aligned} \begin{aligned}&f_{ V_{I}|T_{T},T_{D}<T_{T},R}(v|t_{t},0,r)\int _{0}^{+\infty }f_{T_{I},D'_{2}(s),T_{T},T_{D}<T_{T},R}(t_{i},1,t_{t},0,r)dt_{i}\\&=f_{ V_{I}|T_{T},T_{D}<T_{T},R}(v|t_{t},0,r)\frac{I}{N}f_{T_{T},T_{D}<T_{T},R}(t_{t},0,r)\\&=\frac{I}{N}f_{ V_{I},T_{T},T_{D}<T_{T},R}(v,t_{t},0,r)=\frac{I}{N}P(T_{D}>t_{t}|R=r)f_{T_{T}|R}(t_{t}|r)f_{V_{I}|R}(v|r)f_{R}(r) \end{aligned} \end{aligned}$$

At this point, every density function is known and the double integral can be numerically computed.

### Likelihood contribution of screen detected DCIS

For the DCIS detected individuals, we use *L*(*s*) to denote the size of the DCIS lesion at calendar time point s. We write the exit time from state 2 (counted from the time of entering state 2) as $T_{e}=min\{T_{T},T_{D}\}$ and the inverse of the power law growth function, mapping lesion size to time since entering state 2, as $g_{r}(l)=\frac{r}{k(1-b)}(l^{1-b}-l_{0}^{1-b})>0$. The likelihood distribution for DCIS screen detected individuals can then be written as:

16 $$\begin{aligned} \begin{aligned}&f_{A_{1}(s),L(s)| B(s),\textbf{B}, A(s) }(1,l|1,\textbf{0},1)\propto f_{A_{1}(s),L(s),B(s),\textbf{B}}(1,l,1,\textbf{0})\\&=\int _{0}^{+\infty } P(B(s)=1,\textbf{B}=\textbf{0}|R=r,L(s)=l,A_{1}(s)=1)\Bigg [\int _{g_r(l)}^{+\infty } f_{ T_{e},A_{1}(s),L(s),R}(t_{e},1,l,r)dt_{e}\Bigg ]dr \end{aligned} \end{aligned}$$

Now,

$$\begin{aligned} \begin{aligned}&\int _{g_r(l)}^{+\infty }f_{ T_{e},A_{1}(s),L(s),R}(t_{e},1,l,r)dt_{e}\\&= \int _{g_r(l)}^{+\infty }f_{ L(s)|T_{e},A_{1}(s),R}(l|t_{e},1,r)P(A_{1}(s)=1|T_{e}=t_{e},R=r)f(t_{e},r)dt_{e} \end{aligned} \end{aligned}$$

Using lemmas 1 and 4, the integral above reduces to:

$$\begin{aligned} \begin{aligned}&\int _{g_r(l)}^{+\infty }\frac{1}{t_{e}}g_{r}'(l)\frac{I}{N} t_{e} f_{T_{e}|R}(t_{e}|r)f_{R}(r)dt_{e}\\&= g_{r}'(l)\frac{I}{N}f_{R}(r)P(T_{D}>g_{r}(l)|R=r)P(T_{T}>g_{r}(l)|R=r) \end{aligned} \end{aligned}$$

At this point the single integral likelihood contribution of each screen detected lesion can be numerically computed.

### Likelihood contribution of symptomatically detected DCIS

Finally, for the symptomatically detected individuals, following a framework similar to the derivation of the invasive symptomatic detection likelihood derivation:

$$\begin{aligned} \begin{aligned}&f_{D_{1}(s),L(s)| \textbf{B},D(s) }(1,l|\textbf{0},1)\propto f_{D_{1}(s),L(s),\textbf{B}}(1,l,\textbf{0})\\&= \int _{0}^{+\infty } P(\textbf{B}=\textbf{0}|R=r,L(s)=l,D_{1}(s)=1)f_{L(s)|D'_{1}(s),T_{D}<T_{T},R}(l|1,1,r)\times \\&\quad \times \Bigg [\int _{0}^{+\infty } f_{T_{D},D'_{1}(s),T_{D}<T_{T},R}(t_{d},1,1,r)dt_{d}\Bigg ]dr\\&=\int _{0}^{+\infty } P(\textbf{B}=\textbf{0}|R=r,L(s)=l,D_{1}(s)=1)f_{L_{D}|T_{D}<T_{T},R}(l|1,r)\frac{I}{N}f_{T_{D}<T_{T},R}(1,r)dr \end{aligned} \end{aligned}$$

Where the last equality stems from using the result of theorem 1 for symptomatic DCIS and noting that $f_{L(s)|D'_{1}(s)}(l|1)=f_{L_{D}}(l)$. Then directly, it follows that:

$$\begin{aligned} \begin{aligned}&f_{D_{1}(s),L(s)| \textbf{B},D(s) }(1,l|1,\textbf{0},1)\propto \int _{0}^{+\infty } P(\textbf{B}=\textbf{0}|R=r,L(s)=l,D_{1}(s)=1)f_{L_{D}|R}(l|r)\times \\&\times P(T_{T}>g_{r}(l)|R=r,L_{D}=l)f_{R}(r)dr \end{aligned} \end{aligned}$$

Note that for each likelihood contribution the proportionality constant is either

$$\begin{aligned} \begin{aligned} \frac{I}{NP(B(s)=1,{\textbf {B}}=0,A(s)=1)} \text { or } \frac{I}{NP({\textbf {B}}=0,D(s)=1)} \end{aligned} \end{aligned}$$

depending on whether it is a screen detected (the first) or symptomatic (the second) contribution. Then for each likelihood contribution its proportionality constant can be calculated by solving either for the equation:

$$\begin{aligned} \begin{aligned}&\frac{I}{NP(B(s)=1,{\textbf {B}}=0,A(s)=1)}\\&\quad \times \Bigg [\int _{v_{0}}^{+\infty }f_{A_{2}(s),V(s),B(s),\textbf{B}}(1,v,1,\textbf{0})dv +\int _{l_{0}}^{+\infty }f_{A_{1}(s),L(s),B(s),\textbf{B}}(1,l,1,\textbf{0})dl \Bigg ]=1 \end{aligned} \end{aligned}$$

or for the equation:

$$\begin{aligned} \begin{aligned} \frac{I}{NP({\textbf {B}}=0,D(s)=1)}\Bigg [\int _{v_{0}}^{+\infty }f_{D_{2}(s),V(s),\textbf{B}}(1,v,\textbf{0})dv+\int _{l_{0}}^{+\infty }f_{D_{1}(s),L(s),\textbf{B}}(1,l,\textbf{0})dl \Bigg ]=1 \end{aligned} \end{aligned}$$

## Results

### Confirmation of theoretical results

Before showing how to use the new modelling framework for parameter estimation, we verify through the simulation of a stable disease population under no screening, some of the theoretical results that were derived in Section 2.

#### Simulation of the stable disease population

We simulated the stable disease population based on the parametric assumptions presented in section 2.4. That is, we assumed proportionality of hazards for entering states 3 and 4 from state 2 and that the growth rates of DCIS and invasive cancer are perfectly correlated. We could equally have simulated under non-proportional hazards to demonstrate theoretical results, but for simplicity allow both hazards to be proportional to lesion size, which may in any case be a reasonable assumption.

Parameters $\eta _{D},\eta _{T}$ were selected so that under no screening attendance 90% of the DCIS lesions would transition to invasive cancer before being detected symptomatically. The growth rate distribution was selected to be a Gamma distribution and its parameters were set to be equal to the estimated values from previous studies on invasive breast cancer (Isheden and Humphreys 2022). The value of $\eta _{I}$ was chosen based on the same publication. Although, our choices may not be optimal in terms of how closely they represent the true underlying biological processes, for the purpose of the analysis presented here they suffice; this section aims to mostly verify the theoretical results. All parameter values upon which our simulations are based are shown in table 2.

**Table 2:** Parameter values used for simulating the stable disease population under no screening.

Parameters	$\beta$	*k*	$\tau _{1}$	$\tau _{2}$	$ln(\eta _{T})$	$ln(\eta _{D})$	$ln(\eta _{I})$
Values	-0.8	15	2.36	3	-4.5	-6.5	-8.75

We used the following procedure for generating data. We generated 2000 lesion onsets per (calendar) time point. The time point sequence started from 0, had a step size of 0.01 (which corresponds to 1% of a year) and continued until reaching time point 400. Each lesion was assigned an inverse growth rate from a $G(\tau _1,\tau _2)$ distribution. Based on the inverse growth rate we simulated the times to becoming invasive, $T_{T}$, and the time to having DCIS symptoms, $T_{D}$, and the corresponding lesion sizes at which detection or progression to invasive cancer occurred. If $T_{D}<T_{T}$ the individual would be considered DCIS symptomatic (with time of diagnosis equal to the timepoint of birth plus $T_{D}$, and would never progress to invasive cancer). If $T_{T}<T_{D}$, then, based on the inverse growth rate simulated for that individual, we simulated the time to invasive symptoms $T_{I}$, from which we calculated the clinical tumor volume. Using this simulated dataset, consisting of 200000 onsets per year, we verified the following three theoretical results.

#### Stationary distribution of sizes of invasive cancers

Based on the reduced form of the joint distribution $V(s),A_{2}(s),R,T_{T}$ under proportional hazards, i.e. the result derived as expression (11), we expect that:

17 $$\begin{aligned} \begin{aligned}&f_{d(s)|A_{2}(s)}(d|1)\propto (1.333\pi (0.5d)^{3})'\int _{0}^{+\infty } \int _{0}^{+\infty } f_{V(s),A_{2}(s),R,T_{T}}((1.333\pi (0.5d)^{3}),1,r,t_{t})drdt_{t}\\&\propto (1.333\pi (0.5d)^{3})' \frac{f_{V_{I}}((1.333\pi (0.5d)^{3}))}{(1.333\pi (0.5d)^{3})} \end{aligned} \end{aligned}$$

where *d* is the diameter of the tumor. We note that under proportional hazards the stationary distribution of sizes of invasive cancers is proportional to the corresponding formula presented in Isheden and Humphreys (2022) (under a model for invasive cancer only), but simply has a different proportionality constant.

**Fig. 3:**
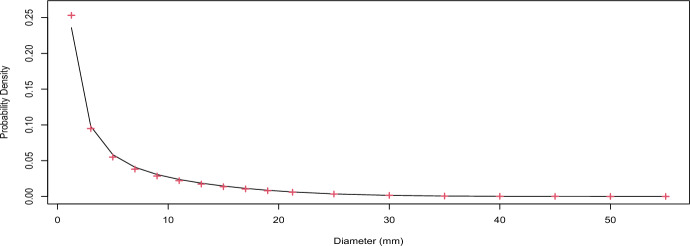
Verification of the stationary distribution of the sizes of latent/preclinical invasive tumors (i.e. the rightmost formula at (17) accounting for the proportionality constant). The solid line represents the theoretical result and the red crosses represent the empirical estimates from the simulation

To verify this result, we selected all preclinical invasive tumors existing at timepoint 340 from the simulated disease population (1554069 invasive tumours). For each of these tumours, based on their assigned growth rate and the time that had passed since it’s onset, we calculated the diameter at timepoint 340. The density $f_{d(340)|A(340)}(d|1)$ was then estimated empirically as the proportion of tumors that had a diameter around different values of *d* and was compared with the analytical formula (the right most expression of (17)). The results, presented in Figure 3, verify the theory.

#### Stationary distribution of sizes of DCIS lesions

Based on the joint distribution of $$ L(s),A_{1}(s),T_{e},R$$ (Sect.4.3) we next verified that:

18 $$\begin{aligned} \begin{aligned}&f_{l(s)|A_{1}(s)}(l|1)\propto \int _{0}^{+\infty }g_{r}'(l)f_{R}(r)P(T_{D}>g_{r}(l)|R=r)P(T_{T}>g_{r}(l)|R=r)dr\\&=k^{-1}l^{-\beta }\mathcal {F}\bigg \{(\eta _{D}+\eta _{T}) \frac{ l^{2-\beta }-l_{0}^{2-\beta } }{k(2-\beta )} \bigg \} \end{aligned} \end{aligned}$$

**Fig. 4:**
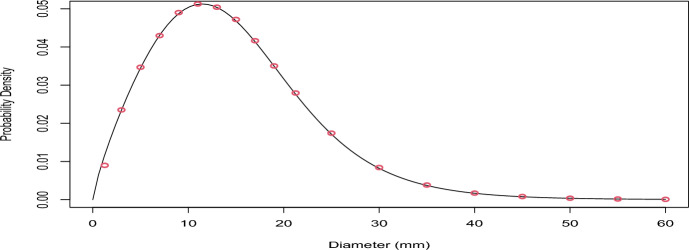
Verification of the stationary distribution of the sizes of latent/preclinical DCIS lesions. The solid line represents the theoretical result and the red circles represent the empirical estimates from the simulation

Using a similarly procedure to that used for invasive cancers, we evaluated the sizes of all of the preclinical DCIS lesions existing at timepoint 340 (in total there were 1069575 preclinical DCIS lesions at this time point). We then compared the empirical density with the analytical formula. The results are presented in Figure 4.

#### Conditional membership probabilities of DCIS lesions and invasive cancers

In this section we essentially verify corollary 2. We empirically estimated the proportion of latent tumors at timepoint 340. We then calculated the proportion based on the analytical formula that was derived. The empirical result of the simulation yielded a 0.4077 proportion of DCIS lesions among preclinical cancers (1069575/(1069575+1554069)). The numerical estimation yielded the same proportion, to four decimal places, showing that the theoretical result holds.

### Estimation of model parameters

We simulated 10 new data sets to assess the estimation of the parameter values based on the likelihood derivations of section 4. We generated data following the same framework presented in 3.1.4 but from timepoint 300 to 340 we also imposed screening examinations every two years. Using the logistic function presented in section 3, we set $b_{0}=-5$ and $b_{2}=0.56$. These choices were based on values estimated from previous studies on invasive breast cancer (Isheden and Humphreys 2022). For the effect of the DCIS lesion in the screening sensitivity we set $b_{1}=0.1$.

**Fig. 5:**
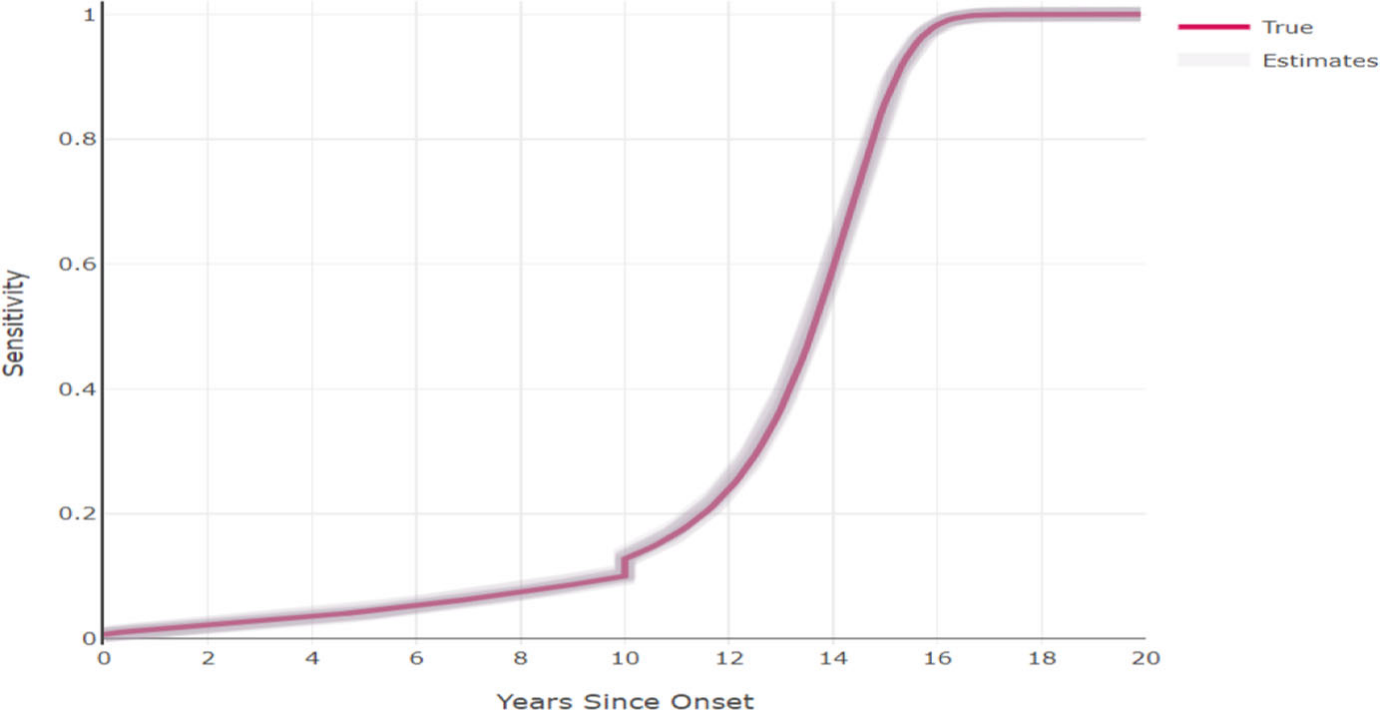
The screening sensitivity function for an individual whose lesion become invasive after 10 years since onset. The function under which data was generated is represented in red and the functions as estimated from the ten simulated data sets are represented as grey lines. The plot is based on an inverse growth rate of 0.67, which is the median growth rate. The discontinuity at 10 years occurs because the smallest tumor volume is considered to be 0.05$\text {mm}^3$

**Fig. 6:**
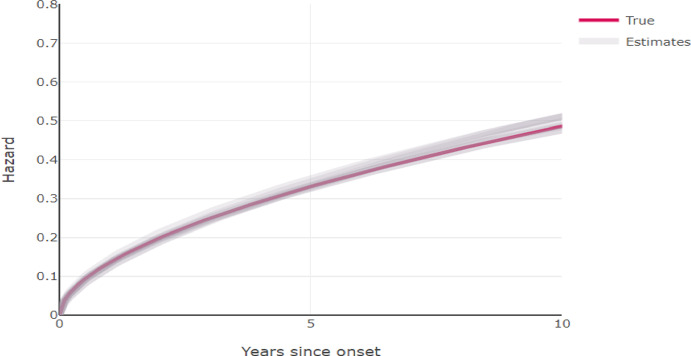
The hazard function for time to progression to invasive cancer. The function under which data was generated is represented in red and the functions as estimated from the ten simulated data sets are represented as grey lines. The plot is based on an inverse growth rate of 0.67, which is the median growth rate in our simulation

For each simulation we sampled a number of cases to which we fitted our likelihood model. The corresponding data for each individual was then their lesion or tumor size (only) at detection and their cancer type (DCIS or invasive cancer), as well as the timepoints of all of their screenings with respect to their diagnosis. Specifically we sampled 14% of the screen detected individuals at timepoint 340, and 14% of the symptomatically detected individuals between the timepoint period 340.00 and 341.99. The percentages of screen detected and symptomatically detected cancers were approximately 80% and 20% respectively. For each simulated data set we estimated the values of 10 parameters. We used the L-BFGS-B algorithm of the optim package in R for the optimization. The starting values of the algorithm were chosen to be a random sample from large range uniform distributions that were centered around the true values of the parameters. The fitted distributions for the screening sensitivity and the hazard function for time to progressing to invasive cancer are presented in figures 5 and 6. Other ways of checking the model fit, e.g. plotting lesion size against time or the screening sensitivity for DCIS or invasive cancer separately with respect to their sizes gave a similar picture, that the estimation could retrieve the underlying process that generate the observed data. Additionally, plotting fitted distributions of lesion/tumor sizes for the specific types of detection against the histograms of the corresponding observed data, yielded good fits (data not shown).

It should be noted that we have selected a flexible growth function for DCIS, i.e. even if we fix *k* to a wrong value, *b* can compensate for our wrong choice and still yield a growth function that is highly similar to the true one. This entails the possibility, that if the starting value for *k* in the optimization is far from its true value, the optimization will converge to a local optimum. The local optimum however, will still provide good estimates for every function that we use, since *b* is the parameter that significantly compensates for the different values of *k*. Additionally, local identifiability issues could occur around the true values of *b* and *k*, since there are many combinations that can yield almost the same growth function. Thus, we generally propose fixing *k* to reasonable values before optimizing the likelihood, even though in this publication we include it in the optimization.

## Discussion

We have developed a mathematical approach which, under the assumption of a stable disease population, can be used to describe the complete natural history of ductal cancer by jointly modeling the processes involved in the evolution and detection of DCIS and its invasive component. By performing simulations based on parametric assumptions found in previous relevant publications, we verified a number of important theoretical results and, further, showed how our model has the potential to be used with observational data to, at a population level, estimate processes of lesion and invasive tumor growth, their detection, and the time it takes for DCIS lesions to progress to invasive cancer. Our approach’s main advantage is that it builds a framework rather than a specific model. Many different distributional assumptions could be made and assessed for each sub-model, for exploring the growth pattern and evolution of DCIS across time.

We acknowledge that in this paper there are limitations induced by our parametric assumptions. For example, there are many reports that a non-negligible proportion of invasive cancers do not result from a DCIS component (i.e. that there exist de novo invasive cancers) (Kole et al. 2019; Chen et al. 2019). Our model could easily be adapted to account for the fact that some cancers will not have an in situ component, by assuming that a proportion of DCIS lesions progress extremely fast to invasive cancer. To address this, a piecewise continuous hazard function, that allows for an initial fast transitioning to invasive breast cancer in a trivially small time interval (e.g. within 0.01 years) could be defined as:

$$ h_{T_{T}|R}(t|r)= \left\{ \begin{array}{ll} a+bt, 0\le t<0.01,\\ \eta _T l_{r}(t-0.01), t\ge 0.01 \end{array} \right. $$

where *a*, *b* satisfy $a+0.01b=0$ (ensuring continuity) and $\exp ( {-\int _{0}^{0.01}h_{T_{T}|R}(t|r)dt}) =1-p$, where *p* corresponds to the proportion of de novo breast cancers. If we consider *p* known and fix it to previously reported values from studies that used large registers containing information on whether the invasive cancer was accompanied by a DCIS lesion (such as Kole et al. (2019) or Chen et al. (2019)), then the parameters *a*, *b* can be directly calculated. In a follow-up paper we plan to fit such a model to data from a large, Swedish, breast cancer screening study. There will be other issues to address. Although the in situ component can indirectly contribute to the detection of invasive tumors, the way we have here incorporated DCIS lesion size in the sensitivity function for invasive cancer, may not be optimal; for observational data, it may significantly inflate the expected number of screen detected cases. Our simulations provide some evidence of this, where many invasive cancers were screen detected because their corresponding DCIS lesions had become very large by the time they had transitioned to invasiveness. Additional approaches, such as assuming that the in situ lesion grows only until it becomes invasive could be considered.

Another assumption in our modeling approach that might not be completely representative of the true breast cancer epidemiology, could be the invariance property of entry, exit and tumor growth distributions through calendar time. In the model we have presented, It is not straightforward to adjust for dependencies between these distributions and calendar time points *s*, since then the population would become unstable and all the properties presented in section 2 would need to be significantly modified. However, we believe that our approximation based on invariant distributions through time is rational enough to accurately estimate parameters of interest in a real world setting. Additionally, if the data is large enough, one can restrict analysis to a relatively short period of time. Theoretically, the distributions need to have been stable only for a length of time that is equal to the maximum time from onset to detection.

Additionally, in Bergholtz et al. (2020) it was suggested that some DCIS lesions may differ in how aggressive they are compared to their invasive components, which implies that in our modeling approach DCIS lesions should have a different growth rate distribution, with notation $R_{D}$. Our framework could also account for such a scenario. The likelihood contributions for invasive cancers would become triple integrals, which would include the density $f_{R_{D}}(r_{d})$. The likelihood form for DCIS lesions would however remain unchanged.

Last, but not least, it may also be important to account for heterogeneity in DCIS growth patterns. In a recent study (Hutten et al. 2023), DCIS tissue was successfully injected into mice and then its time-evolution was investigated. Two main growth patterns were observed: One type of growth involved in situ lesions growing alongside the ducts replacing the normal cells (replacement growth), while the other type involved lesions growing in other directions (expansive growth). The majority of mice that had growths of the second type developed invasive cancer within the study duration while those with replacement growth did not. This finding implies that the lesion diameter might not be a decisive factor for progression to invasive cancer unless there is a certain growth pattern. Thus, where data is available, it may be useful to consider parametric assumptions that indirectly account for the variability in the morphology of growth.

Breast cancer is a highly heterogeneous disease and, based on our framework, however flexible we make our model, we do not expect any set of parametric assumptions to be completely accurate. However, our approach has the potential to use individual-level information from breast cancer patients (size of lesion or tumor, mode of detection, screening pattern, and histopathological covariates in various sub-models) in a coherent way, and gross misspecification should be visible by, for example, plotting the fitted distributions of sizes for each type of detection, or comparing fitted and observed proportions of DCIS/invasive cancers among screen detected and symptomatic cancers. Specific assumptions can of course be compared by using model selection procedures. We, for example, have done this for assumptions about the rates of exiting the preclinical DCIS state. We generated data from a model where the hazard for symptomatic DCIS was proportional to an exponential function of time (and the hazard for pre-clinical invasive was proportional to lesion size), and then fitted two models, one with correct modelling assumptions and one assuming proportional hazards (with both rates proportional to size). The correct model fitted the data (with a sample size of approximately 5000) much better than the misspecified one, with a difference in AIC values of approximately 1900. Also, returning to the comment earlier in this paragraph about strong misspecifications being apparent, we could see that, when fitting the model with proportional hazards to this data, the estimated growth rate distribution of invasive tumours had a density that was almost 0 for R=1 (corresponding to a doubling time of 255 days), compared to the real density in which 50% of tumours have a doubling time longer than 255 days and which approaches 0 at around 1000 days. In a real world setting where there is already prior knowledge on tumor doubling times and the sensitivity of radiologists, misspecification (which in this case is falsely assuming proportionality of hazards) would be very noticeable because the estimated functions would significantly differ from empirical evidence.

At a population level, there remain many challenges to improve understanding of the progression of breast cancer. Here we have laid down a framework and key theoretical results which have the potential to aid exploration of the key underlying processes of breast cancer and its detection.

## Figures and Tables

**Fig. 7:**
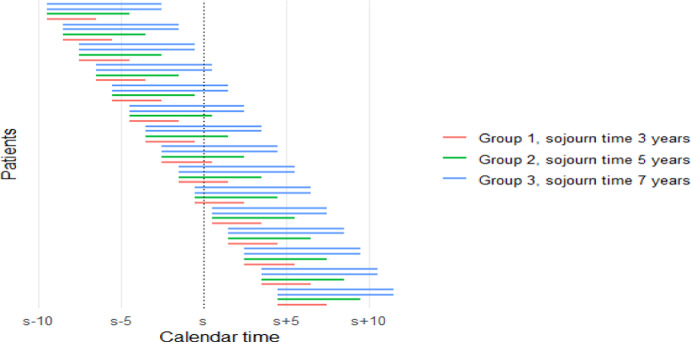
Snapshot of a simple stable disease population showing the sojourn times of 60 individuals that had an onset between $s-10$ and $s+5$. Note that it takes a number of calendar time points for the disease population to become stable

## Data Availability

Not applicable.

## Demonstration of lemma 2 of Isheden and humphreys (2022)

We first show how the result of lemma 2 of Isheden and Humphreys (2022) can be obtained using the terminology and some results of Freeman and Hutchison (1980). We will hereafter refer to the two publications, as IH and FH, respectively. For a 3 state model, i.e. with a single pre-clinical state, lemma 2 of IH stated that $P(A(s)=1|T_{det}=t) \propto t$, where $T_{det}$ is the length of time (the sojourn time) spent in the pre-clinical state. Working under stable disease assumptions and a 3-state model, FH used the following notation

- $I_D$=number of incident cases in unit time with specific total duration *D* (equal to the number of onsets with duration *D* under a stable disease and in the absence of competing events),
- $I_r$=total number of incidence cases per unit time (or per calendar time),
- $P_i(D)$= proportion of cases of a specific duration *D* in an incidence series of cases (which is equal to the proportion of onsets that will have a specific duration *D*), and
- $P_D$=prevalence number of cases (i.e. number of people in the preclinical state with specific duration *D*)

In other words

$$\begin{aligned} P_D=N(\text {prevalent case at a single calendar point and a sojourn time D}) , \end{aligned}$$

where *N*(.) denotes the number of individuals that satisfy the condition inside the parentheses.

Now, based on FH and assuming that $I_r$ is fixed per calendar time, $P_D=P_i(D)DI_r$ (written on p.718 of FH, under the section "Relation between $P_n$ and $I_r$ (12)"). Note that $P_D$ is a number, not a probability. In IH’s notation (their lemma 2) the conditional probability of being in a preclinical state at a particular time point (conditional on sojourn time) is derived, i.e $P(A(s)=1|T_{Det}=t)$. To obtain this conditional probability based on results in FH we then need to divide $P_D$ by a total population size *N* (also assumed to be stable through calendar time) from which the events (cancers in our case) occur, resulting in a probability $P_D/N$.

The selection of what constitutes the "total population" is arbitrary, provided it encompasses a large enough time window (so that the disease population can become stable) and that it is invariant to the calendar timepoints that comprise the time window. For example, *N* can correspond to all the individuals that had their cancer onset within a 20 year interval or the whole population that includes people at risk. Staying with FH notation and using their results, then: (See 7)

$$\begin{aligned} P(\text {prevalent case} | \text {sojourn time D})=\frac{P_D}{NP_i{(D)}}= \frac{I_rD}{N}\propto D \end{aligned}$$

which is the result of lemma 2 of IH, with

$$\begin{aligned} p_i(D)=P(T_{det}=D), P_D=N(A(s)=1,T_{det}=D)\text { and }I_r=I , \end{aligned}$$

mapping the FH notation (left hand side) to our and IH’s notation (right hand side).

To provide a simple illustration of this lemma, we use a simplified and practical example based on discrete time, illustrated in Figure 7. Of course, the lemma is developed with respect to continuous time, but for visualisation it is easier to work with discrete time. Based on the stable disease assumption, we specify a fixed number of onsets during each year (in this case, 4 onsets occurring half way through each year). For illustration, we consider the total population to consist of all individuals that had an onset between $s-10$ and $s+5$, as shown in Figure 7, thus $N=60$. The stable disease assumption also states that the sojourn time distribution of individuals is invariant to the calendar year. In our simplified example, we specify three different sojourn times (3,5 and 7 years) each year, with one occurring with a different frequency to the others (frequencies of 1,1,2, respectively) , implying that $P(T_{det}=7)=0.5$ and $P(T_{det}=5)=P(T_{det}=3)=0.25$.

As can be seen in Figure 7, from the 30 individuals in the population with $T_{det}=7$ years, 14 are preclinical at *s*. The probabilities of being a “prevalent case” at *s* are $\frac{28}{60}$, $\frac{20}{60}$ and $\frac{12}{60}$, for individuals in groups 3, 2 and 1, respectively, as obtained directly as $\frac{I_rD}{N}$ (in FH notation). These probabilities are then $\frac{7}{15}$, $\frac{5}{15}$ and $\frac{3}{15}$ which can be seen to be proportional to sojourn time. Equivalently, illustrating for group 1, in our notation, using the formula $P(A(s)=1|T_{det}=7)$ and expanding it, we get

$$\begin{aligned} \frac{P(A(s)=1,T_{det}=7)}{P(T_{det}=7)}=\frac{N(A(s)=1,T_{det}=7)}{N\times P(T_{det}=7)}=\frac{7\times 4\times P(T_{det}=7)}{60\times P(T_{det}=7)}=\frac{28}{60} \end{aligned}$$

Note how assuming any fixed, but arbitrary and unknown *N* and $I_r$, instead of 60 and 4, and with any *t* for $T_{det}$, we would obtain the result of lemma 2, i.e. $P(A(s)=1|T_{det}=t)\propto t$. We note that this result implies that $P(A(s)=1,T_{det}=t)\propto f_{T_{det}}(t)t$, or using FH notation that $P_D \propto P_i(D)D$.
